# 

**Published:** 1895-08-17

**Authors:** 


### CONTENTS.

The Physiology of Recreation - - - - - - - - 335

The Air of the Schoolroom - - - - - - - - 336

Modern Medico-Psychology and Psychiatry. By T. S. Clouston, M.D.—Criminology - - - - - - - - 337

Health and Athletics - - - - - - - - - - 338

Annotations.—The Fountain Hospital—Radcliffe Infirmary, Oxford—Public Slaughterhouses and Sound Meat - - - - - 339

Notes and News - - - - - - - - - - - 340

Around the Hospitals - - - - - - - - - - 350

Scraps and Gleanings - - - - - - - - - - 350

Medical Progress and Hospital Clinics—

Alcohol in Health and Disease - - - - - - - 341

Progress in Medicine.—Rheumatism and Gout - - - - 342

Progress in Surgery.—Abdominal Surgery - - - - - 344

Progress in Ophthalmology - - - - - - - - - 346

The Institutional Workshop—

Hospital Construction.—West Ham Hospital - - - - 347

Practical Departments - - - - - - - - - 348

Hospital Letters - - - - - - - - - - 349

Construction Notes - - - - - - - - - - 349

### EXTRA SUPPLEMENT.—THE NURSING MIRROR.

News from the Nursing World—In Aid of a Hospital—A Warning to Nurses—Can this be True?—Macclesfield Infirmary—Bradford Medals—An Irish Hospital—Nursing in Aberdeen—Can Six Months' Suffice?—Work in Holland—Our American Sisters—Progress at Baltimore—Women's Work in India—A Successful Ceremony—Short Items - - cxxxv

Elementary Physiology for Nurses. By C. F. Marshall, M.D., B.Sc., F.R.C.S. II.—Introduction (*continued*) cxxxvii

Our Princess's Letter - - - - - - - - cxxxvii

"The Hospital" Convalescent Fund - - - - cxxxvii

Concerning Nurses - - - - - - - - - - cxxxviii
Our American Letter - - - - - - - - - cxxxviii
Minor Appointments - - - - - - - - - cxxxviii
Everybody's Opinion - - - - - - - - - cxxxix
Care of the Sick in Alexandria and Cairo. VI.—The French Hospital - - - - - - - - - - cxxxix
Notes and Queries - - - - - - - - - - cxxxix
Jottings from Murree - - - - - - - - - cxl
For Reading to the Sick - - - - - - - - cxl
Appointments - - - - - - - - - - - - cxl

